# Exploring child and youth understanding of loneliness through qualitative insights and evaluating loneliness measures considering those lived experiences

**DOI:** 10.1111/nyas.15269

**Published:** 2025-01-28

**Authors:** Pamela Qualter, Lily Verity, Wahida Walibhai, Delia Fuhrmann, Laura Riddleston, Iqra Alam, Jasmine Conway, Jennifer Y. F. Lau

**Affiliations:** ^1^ Manchester Institute of Education University of Manchester Manchester UK; ^2^ Psychology Department King's College London London UK; ^3^ Wolfson Centre for Population Health Queen Mary University of London London UK

**Keywords:** assessment tools, experience, loneliness, qualitative methods, young people

## Abstract

The overall goal of this paper is to enhance the understanding and measurement of loneliness by identifying key experiential characteristics of loneliness in children and adolescents, and determine whether there is a need for refined assessment tools that accurately capture that experience. In Study 1, we synthesized the qualitative research on the child and youth experience of loneliness and found shared characteristics of loneliness, with some differences related to developmental changes (e.g., understanding of contexts influencing the experience of loneliness). In Study 2, we reviewed the items from loneliness questionnaires for children and youth and found they do not fully capture the affective dimension of loneliness, that is, the breadth of emotions associated with loneliness. That gap could lead to an incomplete understanding of the phenomenon, potentially undermining the validity of research findings and the effectiveness of interventions designed to alleviate loneliness because they underplay the distress of the experience for children and young people. Addressing this shortcoming should include the development and/or refinement of measurements of loneliness for children and youth, enhancing the accuracy and relevance of loneliness assessments.

## INTRODUCTION

Empirical evidence strongly supports the importance of directly asking people about their experiences to obtain accurate, valid, and comprehensive information.^1^ However, in the field of loneliness, there are few attempts to do that in scale development.^1^, ^2^, ^3^, ^4^, ^5^, ^6^ That means our understanding of how loneliness is experienced relies almost exclusively on researchers’ assumptions or theoretical models. Furthermore, measures of loneliness may be inaccurate and invalid because they do not represent the actual experiences of those with lived experiences of loneliness. In the current study, we first synthesized the recent qualitative work with children (ages 7–10 years) and youth (ages 11–24 years) that explored their experiences of loneliness. We then examined the measures of loneliness used to explore loneliness among children and youth to establish whether items accurately capture the key features of loneliness as described by children and youth.

### The lived experience of loneliness during childhood and adolescence

There are several different conceptualizations of loneliness, but they are all limited in scope, emphasizing certain elements of the loneliness experience over other aspects.^7^ For example, the cognitive discrepancy model^8^ focuses primarily on cognitive aspects of the loneliness experience, with only limited discussion of affective dimensions; the social needs approach^9^ explores the sources of loneliness (the diversity of needs as foundational to loneliness), at the expense of its essence^10^; and the phenomenological approach^11^ highlights how affective states differentiate aspects of the loneliness experience, but cognitive dimensions are overlooked. Those limitations led Stein and Tuval‐Mashiach^7^ to argue for qualitative inquiry that strives to understand the lived experience of loneliness. They argued that qualitative work with people with lived experiences of loneliness was needed to understand the nature of the experience, “raising it to more than an intuitive decision of the researcher” (p. 222).

Using qualitative research methods, including interviews, focus groups, and ethnographic studies, some researchers^12^, ^13^ have explored the nuanced and subjective experiences of loneliness among children and youth. Those studies have captured the depth and complexity of the narratives, allowing for a richer understanding of how loneliness is experienced, perceived, and coped within that demographic.

In 2023, McKenna‐Plumley et al.^14^ published a systematic review and thematic synthesis of 29 studies that investigated the experiences of loneliness in people between 7 and 103 years of age and nonclinical populations. The thematic synthesis highlighted important features of the loneliness experience that were evident across all ages, with 15 descriptive themes that fit under three overarching domains as follows: (1) psychological aspects of the loneliness experience, such as loneliness possessing emotional and cognitive features; (2) interpersonal contextual aspects relating to the role of relationships and interpersonal experiences in loneliness; and (3) personal contextual aspects, including personally challenging experiences contributing to loneliness. Essentially, loneliness for all age groups appears to hinge on the distinction between feelings of meaningful connection and painful disconnection, felt in a general sense or in relation to specific others (e.g., family or peers), and included psychological features (it is painful and distressing). Loneliness was also described by elements of one's personal (social and political) context.

While that review explored different age groups, there were few studies that exclusively explored loneliness among children (*n* = 4) and youth (*n* = 2), making it difficult to determine whether the identified key characteristics of loneliness are crucial to the description of loneliness during childhood and adolescence. However, since the review's publication, there has been an increased number of publications that use qualitative inquiry to examine the experiences of loneliness among young people. Thus, Study 1 explores that new evidence and whether, once combined with findings from previous studies, it fits within the thematic framework that describes the lived experience of loneliness.^14^

### The voices of children and youth in the development of scales to measure loneliness

By capturing subjective experiences and identifying contextual and maintaining factors of loneliness, those qualitative studies offer important information that can advance and refine theoretical frameworks of loneliness; they also play a pivotal role in the development of scales to measure loneliness. The richness and depth of that qualitative data should ensure that the voices of children and youth are heard, and their experiences are comprehensively understood, leading to a more accurate and valid measurement of loneliness for children and youth.

Empirical evidence supports the necessity of incorporating direct input from individuals in the development of questionnaires. It enhances content validity by ensuring the questionnaire comprehensively covers key aspects of the given phenomenon^15^, ^16^ and improves construct validity by ensuring that the questionnaire accurately measures the theoretical construct it intends to assess.^17^, ^18^ Thus, we would expect to see that items on a questionnaire that measures loneliness among children and youth can accurately identify the specific dimensions and manifestations of loneliness that are pertinent to them. Furthermore, they should include context‐specific factors that influence loneliness for people at those ages (i.e., peers, parents, political and social contexts), and developmental understanding of those factors. Incorporating information that comes directly from children and youth with lived experience of loneliness ensures measures of loneliness accurately reflect the subjective experiences of loneliness. Given that few questionnaires achieve that goal,^1^, ^2^, ^3^, ^4^, ^5^, ^6^ in Study 2, we examined whether items of the currently available measures of loneliness that are used with children and youth capture those key features of the experience as they are described by children and youth in Study 1.

### Approach

In Study 1, we performed a systematic review of available published peer‐reviewed and gray literature where young people described their experiences of loneliness. We synthesized the experiences according to the thematic framework developed by McKenna‐Plumley et al.,^14^ and determined whether revisions to the original framework were needed. In Study 2, we explored whether current measures of loneliness used with children and youth accurately capture the important aspects of loneliness as described by them in Study 1.

## STUDY 1

### Methods

We performed a systematic review of all available published peer‐reviewed and the available gray literature that describes young people's experiences of loneliness. Our work builds on that conducted by McKenna‐Plumley et al.^14^ to look specifically at children and youth ages 10–24 years. First, we collated the studies included in McKenna‐Plumley et al.’s synthesis. We conducted the search of academic peer‐reviewed papers and gray literature between January 2023 and July 2023, and again in July 2024 to find other studies that looked at young people's experiences of loneliness; we searched PsycINFO, MEDLINE, Scopus, and Education Resource Information Centre (ERIC) for all studies published on the issue. We also searched the gray literature using Google Scholar, ProQuest Dissertations and Theses, and websites of specific loneliness organizations. We did not apply any restrictions on the publication dates during our literature searches, allowing us to capture a comprehensive range of studies across all relevant time periods. This approach ensured that our review incorporated both historical and contemporary perspectives on the subject matter. Reference lists of included studies were checked, and any relevant studies were harvested from them. A list of all search terms, using the SPIDER tool,^19^ can be found in Table S1. Once duplicates were removed, we used the following inclusion and exclusion criteria to determine which studies would be included in our review.

Inclusion criteria: Only studies with people ages 10–24 years (mean had to be ≤ 24 years where a range of ages are included in the study); only studies that focused on qualitative investigation of experiences of loneliness were included. Exclusion criteria: Studies not meeting the inclusion criteria; studies not published in English; quantitative studies with no qualitative component; studies of clinical populations; studies that reported solely on objective phenomena, such as social isolation rather than the subjectively perceived experience of loneliness.

Once all relevant studies were found, three members of the team (L.V., P.Q., and W.W.) used the McKenna‐Plumley et al. thematic framework to code the experiences detailed in each study. We used the 15 descriptive themes and three overarching domains detailed in that framework^14^ to determine whether key aspects of the loneliness experience originally identified were reported by children and youth. We read the results/findings/discussion sections of each study and used thematic synthesis to code the qualitative data from the various studies and determine whether findings mapped onto the Mckenna‐Plumley et al. framework,^14^ or whether new themes emerged. Coding was completed separately by each author, with agreement on themes reached following discussion; no new patterns or themes were identified.

## RESULTS

We identified 33 studies that explored the experiences of children and youth using qualitative methods. Table 1 shows the qualitative themes across the studies based on McKenna‐Plumley et al.’s thematic framework.^14^ We found evidence for all 15 descriptive themes in the studies that examined the experiences of children and young people; we did not find new themes. There are clear psychological features of loneliness that are experienced across all ages, with people describing loneliness in terms of its distinction from feelings of meaningful connection and painful disconnection. The thematic mapping via theme (Table 1) highlighted variation in the descriptions and understanding of loneliness characterized by age, suggesting there are clear developmental differences. For example, children did not refer to personality or social comparisons as predictors of loneliness, but youth did. Furthermore, it was the case that most studies that included youth with ages ≥ 18 years included mention of the political or societal landscape as predicting loneliness; younger samples were much less likely to include reference to that.

**TABLE 1:** Qualitative themes across studies.

		Psychological aspects				Interpersonal contextual aspects					Personal contextual aspects					
Study	Age of participants in the study	Loneliness is an aversive experience	Loneliness has emotional features	Loneliness has cognitive and perceptual features	Loneliness is impacted by personality and identity	Loneliness can relate to specific relationships (or their absence)	Loneliness related to a lack of close, meaningful relationships—not superficial connections	Loneliness involves feelings of disconnection	Loneliness involves negative interpersonal experiences	Loneliness involves social comparison	Loneliness is connected to, but separate from, aloneness, isolation, and solitude	Loneliness is precipitated by life experiences and transitions	Loneliness fluctuates in duration, intensity, and type	Loneliness can be grounded in specific contexts	Loneliness is impacted by physical and mental health challenges	Loneliness is affected by the sociopolitical landscape
Martin et al.^20^	10–15 years	✓	✓			✓	✓	✓	✓		✓	✓	✓			
Korkiamäki^21^	11–16 years	✓	✓	✓	✓	✓		✓	✓		✓	✓	✓	✓		
Verity et al.^5^	12–18 years	✓	✓	✓	✓	✓	✓	✓	✓	✓	✓	✓		✓	✓	
Verity et al.^6^	14–16 years	✓	✓	✓	✓	✓	✓	✓	✓	✓	✓	✓	✓	✓	✓	
Jenkins et al.^22^	15–17 years	✓	✓			✓	✓	✓	✓	✓	✓	✓	✓	✓	✓	✓
Davidson^13^	15–18 years	✓	✓	✓		✓	✓	✓	✓	✓	✓	✓	✓	✓		
Rew^23^	15–23 years	✓	✓		✓	✓	✓	✓	✓		✓	✓	✓	✓	✓	
Herz and Lalander^24^	15–25 years			✓	✓	✓	✓	✓	✓		✓	✓	✓	✓		✓
Co‐Op Foundation^25^	16–24 years	✓	✓	✓		✓		✓	✓	✓	✓	✓	✓		✓	✓
Sundqvist and Hemberg^26^	17–28 years	✓	✓	✓	✓	✓	✓	✓	✓	✓		✓		✓	✓	✓
Hemberg et al.^27^	17–30 years	✓	✓	✓		✓	✓	✓	✓	✓	✓	✓	✓	✓	✓	✓
Hemberg et al.^28^	17–30 years	✓	✓	✓	✓	✓	✓	✓	✓	✓	✓	✓	✓	✓	✓	✓
Garnow et al.^29^	17.5 years (medium age)	✓	✓	✓	✓	✓	✓	✓	✓	✓	✓	✓			✓	✓
Matthews et al.^30^	18 years			✓	✓	✓			✓			✓		✓	✓	✓
Firmin et al.^31^	18–21 years	✓	✓	✓		✓	✓	✓	✓	✓	✓	✓	✓	✓	✓	
Fardghassemi and Joffe^32^	18–24 years	✓	✓	✓	✓	✓	✓	✓	✓	✓	✓	✓		✓	✓	✓
Wright and Silard^33^	18–25 years	✓	✓	✓	✓	✓	✓	✓	✓	✓	✓	✓	✓	✓		✓
Vasileiou et al.^34^	18–29 years	✓	✓	✓	✓	✓	✓	✓		✓	✓	✓	✓	✓		
Jaud et al.^35^	24.1 (mean age)	✓	✓			✓	✓	✓	✓			✓	✓	✓		✓
Karnick^36^	7–10 years	✓	✓	✓			✓	✓	✓	✓	✓	✓	✓	✓		
Our Voice^37^	7–24 years	✓	✓			✓	✓	✓	✓	✓	✓	✓		✓	✓	✓
Berguno et al.^38^	8–10 years		✓				✓	✓	✓		✓	✓	✓	✓		
Cole et al.^12^	8–10 years	✓	✓	✓		✓	✓	✓	✓				✓	✓		
Kirova‐Petrova^39^	8–10 years	✓	✓	✓		✓	✓	✓	✓	✓	✓	✓		✓	✓	
Kristensen^40^	8–10 years	✓	✓			✓	✓	✓	✓		✓	✓	✓	✓	✓	
Verity et al.^4^	8–14 years	✓	✓	✓	✓	✓	✓	✓	✓	✓	✓	✓	✓	✓		
Cala and Ortega^41^	19–24 years	✓	✓	✓	✓	✓	✓	✓	✓	✓		✓	✓	✓		✓
Garnow et al.^42^	28 years (mean age)	✓	✓	✓	✓	✓	✓	✓	✓	✓	✓	✓	✓			
Hines‐Rome^43^	18–25 years	✓	✓					✓			✓			✓		
Nielsen et al.^44^	18–25 years		✓	✓	✓	✓	✓	✓	✓	✓	✓	✓		✓	✓	✓
Rammya and Kumar^45^	16–24 years	✓				✓		✓	✓						✓	
Turner et al.^46^	13–18 years	✓	✓	✓	✓	✓	✓	✓	✓	✓	✓	✓	✓	✓	✓	✓
Van de Velde et al.^47^	18–30 years	✓	✓	✓		✓	✓	✓		✓	✓	✓	✓	✓	✓	✓
Total count		29	30	24	17	30	28	32	31	23	27	30	23	28	19	16
Percentage of studies (*n* = 33)		87.88	90.91	72.73	51.52	90.91	84.85	96.97	93.94	69.70	81.82	90.91	69.70	84.85	57.58	48.48

*Note*: Garnow et al. (2024) was included despite the mean age of participants exceeded our age range as they were retrospectively describing their experiences during adolescence.^42^ Ticks (✓) in the table indicate that a specific characteristic was supported by the findings in the corresponding study. Blank cells indicate that the study did not provide evidence related to that characteristic or did not report on it.

## STUDY 2

### Methods

We performed a systematic search between January 2023 and July 2023 of the academic literature for measurements (questionnaires and single items) of loneliness used with children or youth ages ≤ 24 years. Search terms are listed in the Table S2. We did not restrict the publication date; the key component of the search was that the authors stated the measure was a measure of loneliness (we also included the term “subjective social isolation”), but not of objective social isolation, social contact, or social support. Once identified, the items from all measures were coded according to the framework developed by McKenna‐Plumley et al.^14^ and supported in Study 1; essentially, each team member noted whether each questionnaire item fit with any of the themes within the framework. That was conducted so we could determine which aspects of loneliness are captured by which measures of loneliness, indicating whether the measures reflect the key characteristics of loneliness as identified by children and youth in Study 1. Coding was completed by all authors. Where more than half the team agreed that the item measured a given dimension of loneliness according to the framework, we noted that as being measured.

## RESULTS

Our search of the literature showed that single‐ (*N* = 2) and multi‐ (*N* = 12) item measures of loneliness have been used to explore the experience among children and/or adolescents. One of the single‐item measures is a direct measure of loneliness because it includes the specific word “lonely.” We found several multi‐item measures of loneliness used to measure loneliness among children and adolescents and those are listed in Table 2. They include the University of California Los Angeles Loneliness Scale (UCLA) and the Children's Loneliness Scale (CLS). Four questionnaires included items that contained the words “lonely” or “loneliness”: CLS, Interpersonal Acceptance‐Rejection Loneliness Scale (IPARLS), Perth A‐Loneness Scale (PALS), and Peer Network and Dyadic Loneliness Scale (PNDLS); but all would be considered indirect measures of loneliness. Both IPARLS and PALS include more than one item that includes the words “lonely” or “loneliness” (IPARLS = 2; PALS = 4), but, as we can see from Table 2, those were the only measures that measured the aversive dimension of the loneliness experience as described by children and youth, and they had used those items referring to “lonely” or “loneliness” to do that.

**TABLE 2:** Key features of the loneliness experience examined in existing loneliness measures.

					Qualitative themes														
					Psychological aspects				Interpersonal contextual aspects					Personal contextual aspects					
Measure	Response instructions	Number of items in measure	Number of items screened	Number of key features examined through the screened items	An aversive experience	Emotional features	Cognitive and perceptual features	Impacted by personality and identity	Relates to specific relationships (or their absence)	A lack of close, meaningful relationships—not superficial connections	Feelings of disconnection/connection	Negative interpersonal experiences	Social comparison	Connected to, but separate from, aloneness, isolation, and solitude	Precipitated by life experiences and transitions	Fluctuates in duration, intensity, and type	Grounded in specific contexts	Impacted by physical and mental health challenges	Affected by the sociopolitical landscape
Children's Loneliness Scale (CLS)^48^	Not specified.	16	8	6			✓		✓	✓	✓	✓		✓					
Differential Loneliness Scale (DLS)^49^	For each statement, decide whether it describes you or your situation or not.	60	28	9		✓	✓	✓	✓	✓	✓	✓			✓				✓
Three‐Item Loneliness Scale^50^	The next questions are about how you feel about different aspects of your life. For each one, tell me how often you feel that way.	3	3	6			✓		✓	✓	✓	✓		✓					
Interpersonal Acceptance‐Rejection Loneliness Scale (IPARLS)^51^	Not specified.	15	15	9	✓	✓	✓		✓	✓	✓	✓	✓	✓					
Loneliness and Aloneness Scale for Children and Adolescents (LACA)^52^	Not specified.	48	17	8		✓	✓		✓	✓	✓	✓		✓			✓		
Perth A‐Loneness Scale (PALs)^53^	Not specified.	24	24	7	✓	✓	✓		✓	✓	✓			✓					
Peer Network and Dyadic Loneliness Scale (PNDLS)^54^	Participants are first asked to select which of the two types of children they are most like and then to specify whether the chosen description is sort of true or really true for them.	16	14	7			✓	✓	✓	✓	✓	✓	✓						
Rotenberg 3‐item Loneliness and Social Dissatisfaction Measure^55^	In the last 2 weeks, to what extent have you felt the following.	3	3	6			✓		✓	✓	✓	✓		✓					
Relational Provisions Loneliness Questionnaire (RPLQ)^56^	Participants rate how true each statement is for them.	28	27	5			✓	✓	✓	✓	✓								
Rasch‐Type Loneliness Scale (RTLS)/De Jong Gierveld Loneliness Scale (DJGLS)^57^	Please indicate for each of the statements, the extent to which they apply to your situation, the way you feel now.	11	10	6		✓	✓		✓	✓	✓	✓							
Social and Emotional Loneliness Scale for Adults (SELSA)^58^	Participants are asked to indicate the degree of agreement or disagreement with each statement.	37	30	6			✓	✓	✓	✓	✓			✓					
Single item^59^	Not specified.	1	1	1							✓								
Single item^60^	Response options: 0 = “no, does not apply,” 1 = “yes, it applies, but I do not suffer from it,” 2 = “yes, it applies, and I suffer slightly,” 3 = “yes, it applies, and I suffer moderately,” 4 = “yes, it applies, and I suffer strongly.”	1	1	2					✓					✓					
University of California Los Angeles (UCLA) Loneliness Scale^61^	Indicate how often you feel the way described in each of the following statements.	20	18	6			✓	✓	✓	✓	✓			✓					

*Note*: The CLS contains a total of 24 items, eight of which are filler items. The items screened in the CLS, DLS, LACA, PNDLS, RPLQ, RTLS, SELSA, and UCLA Loneliness Scale were those rated as measuring loneliness.^3^ Where more than half the team agreed that the item measured a given dimension of loneliness according to the framework, we noted that as being measured, and that is represented by a tick (✓) in the table.

Coding all items from all measures was completed according to the framework developed by McKenna‐Plumley et al.^14^ and supported in Study 1. All authors completed that task, with agreed, overall findings noted in Table 2 for each measure; item analyses for each measure can be found in Table S3.

We found that the current measures of loneliness used in research with children and adolescents often fail to encompass the multifaceted nature of loneliness as determined by the framework; some measures are better than others. The single‐item measures are unable to capture the full spectrum of features specific to the experience of loneliness, but other measures also fall short by not asking about the essential psychological features of loneliness as described by children and youth (the aversive, distressing nature of the experience); all measures, except for the single‐item measurements, highlight the cognitive and perceptual features of loneliness (it was related to one's perceptions, to behavior, and/or to existential concerns about life and belonging), the fact that loneliness can relate to specific experiences, and that it is distinct from feelings of meaningful connection.

## DISCUSSION

There are several key findings of the current research. First, the thematic framework developed by McKenna‐Plumley et al.^14^ proved successful for further synthesizing the experience of loneliness among children and youth; no new additional information needed to be incorporated into the model. Once the extant literature was synthesized, we showed that loneliness manifests with distinct psychological characteristics that are universally experienced across childhood and youth ages 10–24 years, with individuals often describing it in terms of lacking meaningful connections and feeling a sense of disconnection. There are clear developmental differences too. Second, we found that questionnaires used to measure loneliness among children and youth are limited because they do not fully capture the experience of loneliness as discussed by children and youth. Indeed, the defining characteristic that loneliness is a distressing, unpleasant experience is missing from almost all measures. This likely comes from the fact that the measures have not been developed based on the primary qualitative exploration of the construct and through discussion with children and/or youth.

Our examination of the qualitative literature of child and youth loneliness highlights the universal psychological dimensions of loneliness, characterized by its contrast with meaningful connection and the distress of disconnection; when synthesized, we also see that there are developmental distinctions in the experience of loneliness. Consistent with developmental changes in social cognition,^62^ which involves perceiving, interpreting, and understanding the behavior, intentions, and emotions of both ourselves and others, our qualitative synthesis shows that children and youth's abilities to navigate and interpret social relationships directly impact how they experience and understand loneliness. Children, for instance, do not appear to attribute loneliness to personality traits or social comparisons; in contrast, those are viewed by adolescents and emerging adults as prominent predictors in the experience of loneliness and form a clear part of their conversations about that experience. Why such attributions are not found for children, but for youth needs to be explored in future work, but it likely comes about due to increased development of socioemotional skills. Furthermore, studies involving children and youth up to 18 years of age reveal that political or societal contexts are not commonly cited as factors influencing loneliness; in contrast, older youth frequently referenced political and societal landscapes as contributory elements, and those constitute a significant aspect of their discourse regarding the experience of loneliness. Thus, older youth (≥ 18 years of age) discussed loneliness as being impacted by social and cultural norms, the availability of resources and services, and referencing discrimination. It is also in samples that include older youth where discussion of the link between loneliness and (mental and physical) health is discussed; such descriptions of the experience were absent from conversations with children and younger youth. Given our findings, we urge researchers, educators, and those developing interventions to recognize how the experience and contributing factors of loneliness change with age, customizing support as needed. For older youth, discussions and support mechanisms that consider the influence of societal and political contexts on their experience of loneliness should be considered; encouraging civic engagement and community involvement is likely to help mitigate those factors.

In the qualitative studies synthesized in Study 1, children and youth made reference to the emotional aspect of loneliness, but reference to loneliness as an emotional experience (it is painful and distressing) is missing from most measures that are used in research exploring loneliness during childhood and adolescence; there is a focus on behavioral dimensions (i.e., “I feel left out of things” [CLS], “my friends don't seem to stay interested in me for long” [DLS]). One measure (IPARLS) does very well on this dimension, with inquiries (“It bothers me that I am so isolated,” and “It hurts to be so alone”) that link specific experiences with distress felt by the child or youth. The fact that inquiries addressing loneliness as distressing are notably absent from most questionnaires concerning loneliness in children and youth poses significant problems for research validity and intervention efficacy.^a^ Specifically, an incomplete understanding of the emotional impact of loneliness potentially results in the development of interventions and treatments that do not fully address the emotional and psychological needs of individuals. Such oversight might impede the effectiveness of strategies designed to mitigate loneliness and could hinder efforts to accurately assess the severity and implications of loneliness on mental health.

## CONCLUSION

The omission of key dimensions of loneliness as highlighted by children and youth during qualitative work is significant because it suggests that existing measures do not fully capture the affective dimension of loneliness. That likely comes from the fact that the measures do not appear to have been generated based on primary qualitative exploration of the construct and through discussion with children and/or youth. Thus, a crucial part of scale development to inform domains and items^63^, ^64^ has been missing from the development of loneliness measures; that finding does not appear to be specific to measurements of loneliness for children and youth,^3^ but extends to other age groups too. Given that gap could lead to an incomplete understanding of the phenomenon, potentially undermining the validity of research findings and the effectiveness of interventions designed to alleviate loneliness, we call for the development of new measures of loneliness and/or refinement of current ones so that they accurately capture authentic loneliness experiences.

## AUTHOR CONTRIBUTIONS

P.Q., J.L., and L.V. conceived the study; P.Q. and L.V. participated in its design and coordination. L.V., W.W., and P.Q. searched and screened data for Study 1; L.V. and W.W. conducted searches for Study 2; P.Q., L.V., W.W., D.F., L.R., I.A., J.C., and J.Y.F.L. coded for Study 2. W.W. and P.Q. managed the curation of data for Study 1; L.V. managed the curation of data for Study 2. P.Q. drafted the manuscript. L.V., W.W., D.F., L.R., I.A., J.C., and J.Y.F.L. commented on versions of the full manuscript. All authors read and approved the final manuscript. P.Q. ensured data were made available as Supplementary Material.

## COMPETING INTERESTS

The authors report no conflict of interest.

### PEER REVIEW

The peer review history for this article is available at https://publons.com/publon/10.1111/nyas.15269.

## Supporting information



Supporting Information

## Footnotes

a We also considered that the statements in questionnaires introducing the items to participants may have asked them to consider items in light of their assertive nature. Table S3 shows that is not the case.
