# Supplementary material for: Exploring child and youth understanding of loneliness through qualitative insights and evaluating loneliness measures considering those lived experiences
Source: Ann N Y Acad Sci. 2025 Jan 28;1544(1):42–54. doi: 10.1111/nyas.15269 (PMC11829323; doi:10.1111/nyas.15269)
Supplement: Supplementary file 1 — Supporting Information [file NYAS-1544-42-s001.docx]

**Inside the Lonely Young Mind: Exploring Youth Experiences through Qualitative Insights**

Pamela Qualter^1^, Lily Verity^1^, Wahida Walibhai^1^, Delia Fuhrmann^2^, Iqra Alam^3^, Jasmine Conway^1^, Jennifer Y. F. Lau^3^

1. Manchester Institute of Education, University of Manchester.
2. Psychology Department, King’s College London.
3. Wolfson Centre for Population Health, Queen Mary University of London

**Supporting Information**

**Study 1 Supporting Information**

**SPIDER** tool^19^ (Sample, Phenomenon of Interest, Design, Evaluation) was used as follows:

**S** Individuals younger than 24 years who describe experiences of loneliness. Does not include specific clinical populations

**PI** Loneliness

**D** Any qualitative research design e.g. interview, focus group, etc. mixed methods designs that

include qualitative methods are included if the qualitative findings are reported separately.

**E** Descriptions of experiences of loneliness

**R** Primary qualitative research

**Inclusion Criteria**

- Only studies with people ages younger than 24 years will be included. Databases will be searched from date of inception.
- Studies which focus on qualitatively studying experiences of loneliness will be included.

**Exclusion Criteria**

1. Studies not meeting the inclusion criteria (defined by SPIDER)

2. Studies not published in English

3. Quantitative studies with no qualitative component

4. Studies of clinical populations

5. Studies that report solely on objective phenomena such as social isolation rather than the

subjectively perceived experience of loneliness

6. Studies where the primary focus is on experiences of loneliness. That is defined as studies where those experiences are a key aspect (such as a central aim/objective) of the work rather than simply a part of the output.

**Other Useful Information.** Exploring loneliness as a subjectively experience that is separate from objective experiences such as socially isolation, social support, and social connection. Following McKenna-Plumley et al., we are interested only in synthesising qualitative research that focuses on subjective experiences of loneliness in non-clinical populations. Different to McKenna-Plumley et al., we are only interested in the experience of youth (people younger than 24 years).

**Table S1**. Search terms used in Study 1 to explore experiences of loneliness among youth.

| **Sample: young people** |
| --- |
| 1 child* |
| 2. adolescen* |
| 3. youth |
| 4. emerging adult* |
| 5. young adult* |
| **Experience: Loneliness** |
| 6. lonel* |
| 7. combine 6 with 1, 2, 3, 4, 5, with OR |
| **Experience: Qualitative Research** |
| 8. qualitative* |
| 9. "mixed method*" |
| 10. "mixed-method*" |
| 11. “interview* |
| 12. "focus group* |
| 13. "thematic analysis" |
| 14. "narrative analysis" |
| 15. "narrative approach" |
| **Method: Qualitative** |
| 16. "grounded theory" |
| 17. "phenomenological analysis" |
| 18. "phenomenological approach" |
| 19. ethnograph* |
| 20. "discourse analysis" |
| 21. "content analysis" |
| 22. "lived experience" |
| 23. "group discussion" |
| 24. "case stud*" |
| 25. combine 7 AND 8, 9, 10, 11, 12, 13, 14, 15, 16, 17, 18, 19, 20, 21, 22, 23, 24 with OR |

**Study 2 Supporting Material**

**SPIDER** tool^19^ (Sample, Phenomenon of Interest, Design, Evaluation) was used as follows:

**S** Individuals younger than 24 years. Does not include specific clinical populations

**PI** Loneliness

**D** Any quantitative research design, that includes analyses of data from questionnaires that measure loneliness

**E** Uses measures of loneliness

**R** Primary quantitative research

**Inclusion Criteria**

- Only studies with people ages younger than 24 years will be included. Databases will be searched from date of inception.
- Studies which focus on quantitative study of loneliness during childhood or youth (ages 10-24 years).

**Exclusion Criteria**

1. Studies not meeting the inclusion criteria (defined by SPIDER)

2. Studies not published in English

3. Qualitative studies with no quantitative component

4. Studies of clinical populations

5. Studies that report solely on objective phenomena such as social isolation rather than the

subjectively perceived experience of loneliness

**Table S2. Search terms for Study 2.**

|  |
| --- |
| **Sample: young people** |
| 1 child* |
| 2. adolescen* |
| 3. youth |
| 4. emerging adult* |
| 5. young adult* |
| **Experience: Loneliness** |
| 6. lonel* |
| 7. combine 6 WITH 1, 2, 3, 4, 5, with OR |
| **Experience: Quantitative Research** |
| 8. quantitative* |
| 9. "mixed method*" |
| 10. "mixed-method*" |
| **Method: Quantitative** |
| 11. “survey* |
| 12. "questionnaire* |
| 13. Combine 7 AND 8, 9, 10, 11, 12, with OR |

**Supporting Table S3**

Key Features of the Loneliness Experience Examined in Individual Items in Loneliness Measures

| Measure | Authors | Response Instructions | Item | Key Features of the Loneliness Experience | | | | | | | | | | | | | | |
| --- | --- | --- | --- | --- | --- | --- | --- | --- | --- | --- | --- | --- | --- | --- | --- | --- | --- | --- |
|  |  |  |  | An aversive experience | Emotional features | Cognitive and perceptual features | Impacted by personality and identity | Relates to specific relationships (or their absence) | A lack of close, meaningful relationships- not superficial connections | Feelings of disconnection/ connection | Negative interpersonal experiences | Social comparison | Connected to, but separate from, aloneness, isolation, and solitude | Precipitated by life experiences and transitions | Fluctuates in duration, intensity, and type | Grounded in specific contexts | Impacted by physical and mental health challenges | Affected by the socio-political landscape |
| Children's Loneliness Scale (CLS)^48^ | Asher et al. (1984) | Options: always true, true most of the time, sometimes true, hardly ever true, not true at all. | I am lonely |  |  | ✓ |  |  |  | ✓ |  |  |  |  |  |  |  |  |
|  |  |  | I can find a friend when I need one |  |  |  |  | ✓ |  |  |  |  |  |  |  |  |  |  |
|  |  |  | I don't have any friends |  |  |  |  | ✓ |  |  |  |  |  |  |  |  |  |  |
|  |  |  | I don't have anyone to play with |  |  |  |  | ✓ |  |  |  |  |  |  |  |  |  |  |
|  |  |  | I feel alone |  |  | ✓ |  |  |  | ✓ |  |  | ✓ |  |  |  |  |  |
|  |  |  | I feel left out of things |  |  | ✓ |  |  |  | ✓ | ✓ |  |  |  |  |  |  |  |
|  |  |  | I have nobody to talk to |  |  |  |  | ✓ | ✓ | ✓ |  |  |  |  |  |  |  |  |
|  |  |  | There's nobody I can go to when I need help |  |  |  |  |  | ✓ |  |  |  |  |  |  |  |  |  |
| Differential Loneliness Scale (DLS)^49^ | Schmidt & Sermat (1983) | For each statement, decide whether it describes you or your situation or not.  Options: true or false. | A lot of my friendships ultimately turn out to be pretty disappointing |  |  |  |  | ✓ | ✓ |  |  |  |  |  |  |  |  |  |
|  |  |  | Few of my friends understand me the way I want to be understood |  |  | ✓ |  | ✓ | ✓ | ✓ |  |  |  |  |  |  |  |  |
|  |  |  | I can't depend on getting moral, or financial support from any group or organisation in a time of trouble |  |  |  |  | ✓ |  | ✓ |  |  |  | ✓ |  |  |  | ✓ |
|  |  |  | I don't feel that I can turn to my friends living around me for help when I need it |  |  |  |  | ✓ | ✓ |  |  |  |  |  |  |  |  |  |
|  |  |  | I don't get much satisfaction from the groups I attend |  |  |  |  | ✓ | ✓ |  |  |  |  |  |  |  |  |  |
|  |  |  | I don't have any neighbours who would help me out in a time of need |  |  |  |  | ✓ | ✓ |  |  |  |  |  |  |  |  |  |
|  |  |  | I don't have any one special love relationship in which I feel really understood |  |  | ✓ |  | ✓ | ✓ |  |  |  |  |  |  |  |  |  |
|  |  |  | I don't think that anyone in my family really understands me |  |  | ✓ |  | ✓ | ✓ | ✓ |  |  |  |  |  |  |  |  |
|  |  |  | I get plenty of help and support from friends |  |  |  |  | ✓ | ✓ |  |  |  |  |  |  |  |  |  |
|  |  |  | I have a lover or spouse who fulfils many of my emotional needs |  |  |  |  | ✓ | ✓ |  |  |  |  |  |  |  |  |  |
|  |  |  | I have at least one good friend of the same sex |  |  |  |  | ✓ | ✓ |  |  |  |  |  |  |  |  |  |
|  |  |  | I have at least one real friend |  |  |  |  | ✓ | ✓ |  |  |  |  |  |  |  |  |  |
|  |  |  | I have few friends that I can depend on to fulfil their end of mutual commitments |  |  |  |  | ✓ | ✓ |  | ✓ |  |  |  |  |  |  |  |
|  |  |  | I have few friends with whom I can talk openly |  |  |  |  | ✓ | ✓ |  |  |  |  |  |  |  |  |  |
|  |  |  | I really feel that I belong to a family |  |  |  |  | ✓ |  |  |  |  |  |  |  |  |  |  |
|  |  |  | I seldom get the emotional security I need from a romantic or sexual relationship |  | ✓ |  |  | ✓ | ✓ |  |  |  |  |  |  |  |  |  |
|  |  |  | Members of my family give me the kind of support that I need |  |  |  |  | ✓ | ✓ |  |  |  |  |  |  |  |  |  |
|  |  |  | Most everyone around me is a stranger |  |  |  |  |  | ✓ | ✓ |  |  |  |  |  |  |  |  |
|  |  |  | Most of my friends are genuinely concerned about my welfare |  |  |  |  | ✓ | ✓ |  |  |  |  |  |  |  |  |  |
|  |  |  | Most of my friends understand my motives and reasoning |  |  |  | ✓ | ✓ | ✓ |  |  |  |  |  |  |  |  |  |
|  |  |  | My friends are generally interested in what I am doing, although not to the point of being nosey |  |  |  |  | ✓ | ✓ |  |  |  |  |  |  |  |  |  |
|  |  |  | My friends don't seem to stay interested in me for long |  |  | ✓ |  | ✓ | ✓ |  | ✓ |  |  |  |  |  |  |  |
|  |  |  | My romantic or marital partner gives me much support and encouragement |  |  |  |  | ✓ | ✓ |  |  |  |  |  |  |  |  |  |
|  |  |  | No one in the community where I live cares much about me |  |  |  |  | ✓ | ✓ | ✓ |  |  |  |  |  |  |  |  |
|  |  |  | People in my community aren't really interested in what I think or feel |  |  |  | ✓ | ✓ | ✓ | ✓ |  |  |  |  |  |  |  |  |
|  |  |  | Right now, I don’t have true compatibility in a romantic or marital relationship |  |  |  |  | ✓ | ✓ |  |  |  |  |  |  |  |  |  |
|  |  |  | Some of my friends will stand by me in almost any difficulty |  |  |  |  | ✓ | ✓ |  |  |  |  |  |  |  |  |  |
|  |  |  | There are people in my community who understand my views and beliefs |  |  |  | ✓ | ✓ | ✓ |  |  |  |  |  |  |  |  | ✓ |
| Three-Item Loneliness Scale^50^ | Hughes et al. (2004) | The next questions are about how you feel about different aspects of your life. For each one, tell me how often you feel that way.  Options: Hardly ever (1), some of the time (2), often (3). | How often do you feel isolated from others? |  |  |  |  | ✓ |  | ✓ |  |  | ✓ |  |  |  |  |  |
|  |  |  | How often do you feel left out? |  |  |  |  |  |  | ✓ | ✓ |  |  |  |  |  |  |  |
|  |  |  | How often do you feel that you lack companionship? |  |  | ✓ |  | ✓ | ✓ |  |  |  |  |  |  |  |  |  |
| Interpersonal Acceptance-Rejection Loneliness Scale (IPARLS)^51^ | Rohner & Molaver (2015) | Options: “Almost Never True” (1), “Not Often True” (2), “Sometimes True” (3), “Often True” (4), “Almost Always True” (5). | I am distracted by feelings of loneliness | ✓ | ✓ | ✓ |  |  |  |  |  |  |  |  |  |  |  |  |
|  |  |  | I am unhappy because I am not part of a social group |  | ✓ |  |  | ✓ |  | ✓ |  |  |  |  |  |  |  |  |
|  |  |  | I am unhappy because too many others view me with indifference |  | ✓ | ✓ |  |  |  | ✓ | ✓ |  |  |  |  |  |  |  |
|  |  |  | I could really use the company of others |  |  |  |  |  |  | ✓ |  |  |  |  |  |  |  |  |
|  |  |  | I feel bad because I am isolated from others | ✓ | ✓ |  |  | ✓ |  | ✓ |  |  |  |  |  |  |  |  |
|  |  |  | I feel dejected because my circle of friends is too limited | ✓ | ✓ | ✓ |  | ✓ |  |  |  |  |  |  |  |  |  |  |
|  |  |  | I feel like reaching out to others so I won't feel so alone |  |  | ✓ |  |  |  | ✓ |  |  | ✓ |  |  |  |  |  |
|  |  |  | I feel lonely |  |  | ✓ |  |  |  | ✓ |  |  |  |  |  |  |  |  |
|  |  |  | I feel sad because I don't have companionship | ✓ | ✓ |  |  | ✓ | ✓ |  |  |  |  |  |  |  |  |  |
|  |  |  | I feel unhappy because I am left out | ✓ | ✓ |  |  | ✓ |  | ✓ | ✓ |  |  |  |  |  |  |  |
|  |  |  | I have a sense of emptiness because I lack friends | ✓ | ✓ |  |  | ✓ |  |  |  |  |  |  |  |  |  |  |
|  |  |  | I wish I had as many friends as other people |  |  | ✓ |  | ✓ |  | ✓ |  | ✓ |  |  |  |  |  |  |
|  |  |  | I wish I had more friends |  |  | ✓ |  | ✓ |  |  |  |  |  |  |  |  |  |  |
|  |  |  | It bothers me that I am so isolated | ✓ | ✓ |  |  |  |  | ✓ |  |  | ✓ |  |  |  |  |  |
|  |  |  | It hurts to be so alone | ✓ | ✓ |  |  |  |  |  |  |  | ✓ |  |  |  |  |  |
| Loneliness and Aloneness Scale for Children and Adolescents (LACA)^52^ | Marcoen et al. (1987) | Options: often, sometimes, seldom, never. | I am afraid that others won't let me join in |  | ✓ | ✓ |  |  |  | ✓ | ✓ |  |  |  |  |  |  |  |
|  |  |  | I feel isolated from other people |  |  |  |  |  |  | ✓ |  |  |  |  |  |  |  |  |
|  |  |  | I feel abandoned by my friends |  |  | ✓ |  | ✓ |  |  | ✓ |  |  |  |  |  |  |  |
|  |  |  | I feel alone at school |  |  |  |  | ✓ |  | ✓ |  |  | ✓ |  |  | ✓ |  |  |
|  |  |  | I feel excluded by my classmates |  |  |  |  | ✓ |  | ✓ |  |  |  |  |  | ✓ |  |  |
|  |  |  | I feel I have very strong ties with my parents |  |  |  |  | ✓ | ✓ |  |  |  |  |  |  |  |  |  |
|  |  |  | I feel left out by my friends |  |  | ✓ |  | ✓ |  |  | ✓ |  |  |  |  |  |  |  |
|  |  |  | I feel left out by my parents |  |  |  |  | ✓ |  | ✓ | ✓ |  |  |  |  |  |  |  |
|  |  |  | I feel sad because I have no friends |  | ✓ |  |  | ✓ | ✓ |  |  |  |  |  |  |  |  |  |
|  |  |  | I feel sad because nobody wants to join in with me |  | ✓ |  |  | ✓ |  | ✓ | ✓ |  |  |  |  |  |  |  |
|  |  |  | I find consolation with my parents |  |  |  |  | ✓ | ✓ |  |  |  |  |  |  |  |  |  |
|  |  |  | I have the feeling that my parents and I belong together |  |  |  |  | ✓ | ✓ | ✓ |  |  |  |  |  |  |  |  |
|  |  |  | I think there is no single friend whom I can tell everything |  |  | ✓ |  | ✓ | ✓ |  |  |  |  |  |  |  |  |  |
|  |  |  | I want to be better integrated in the class group |  |  |  |  | ✓ |  | ✓ | ✓ |  |  |  |  |  |  |  |
|  |  |  | My parents are ready to listen to me or to help me |  |  |  |  | ✓ | ✓ |  |  |  |  |  |  |  |  |  |
|  |  |  | My parents make time to pay attention to me |  |  |  |  | ✓ | ✓ |  |  |  |  |  |  |  |  |  |
|  |  |  | My parents show real interest in me |  |  |  |  | ✓ | ✓ |  |  |  |  |  |  |  |  |  |
| Perth A-Loneness Scale (PALs)^53^ | Houghton et al. (2014) | Options: never, rarely, sometimes, often, very often, always. | I am not close to anyone |  |  |  |  | ✓ | ✓ | ✓ |  |  |  |  |  |  |  |  |
|  |  |  | I am unhappy being so isolated from others |  | ✓ |  |  |  |  | ✓ |  |  |  |  |  |  |  |  |
|  |  |  | I can turn to my friends to help when I need it |  |  |  |  | ✓ | ✓ |  |  |  |  |  |  |  |  |  |
|  |  |  | I do not have a close friend |  |  |  |  | ✓ | ✓ |  |  |  |  |  |  |  |  |  |
|  |  |  | I feel calm and relaxed when by myself | ✓ | ✓ |  |  |  |  |  |  |  | ✓ |  |  |  |  |  |
|  |  |  | I feel happy when I'm all by myself | ✓ | ✓ |  |  |  |  |  |  |  | ✓ |  |  |  |  |  |
|  |  |  | I feel like I do not have a friend in the world |  |  | ✓ |  | ✓ | ✓ | ✓ |  |  |  |  |  |  |  |  |
|  |  |  | I feel part of a group of friends |  |  |  |  | ✓ |  | ✓ |  |  |  |  |  |  |  |  |
|  |  |  | I feel sad because I have no friends | ✓ | ✓ |  |  | ✓ |  |  |  |  |  |  |  |  |  |  |
|  |  |  | I get plenty of help and support from friends |  |  |  |  | ✓ | ✓ |  |  |  |  |  |  |  |  |  |
|  |  |  | I have discovered the benefits of being alone |  |  |  |  |  |  |  |  |  | ✓ |  |  |  |  |  |
|  |  |  | I have friends that I can trust to do what they say they will do |  |  |  |  | ✓ | ✓ |  |  |  |  |  |  |  |  |  |
|  |  |  | I have nobody to talk to |  |  |  |  | ✓ | ✓ | ✓ |  |  |  |  |  |  |  |  |
|  |  |  | I want to be alone |  |  |  |  |  |  |  |  |  | ✓ |  |  |  |  |  |
|  |  |  | If I feel lonely I don't know what to do | ✓ |  |  |  |  |  |  |  |  | ✓ |  |  |  |  |  |
|  |  |  | Most of my friends are true friends |  |  |  |  | ✓ | ✓ |  |  |  |  |  |  |  |  |  |
|  |  |  | My friends will stand by me in almost any difficulty |  |  |  |  | ✓ | ✓ |  |  |  |  |  |  |  |  |  |
|  |  |  | No one cares much about me |  |  | ✓ |  |  | ✓ |  |  |  |  |  |  |  |  |  |
|  |  |  | There are benefits of being on my own |  |  |  |  |  |  |  |  |  | ✓ |  |  |  |  |  |
|  |  |  | There are positive things about being lonely | ✓ |  |  |  |  |  |  |  |  | ✓ |  |  |  |  |  |
|  |  |  | When I am all by myself, I wish I had a friend to be with |  |  |  |  | ✓ |  |  |  |  |  |  |  |  |  |  |
|  |  |  | When I am by myself I feel lonely |  |  |  |  |  |  |  |  |  | ✓ |  |  |  |  |  |
|  |  |  | When I am lonely time seems to drag and I don't enjoy things | ✓ |  | ✓ |  |  |  |  |  |  | ✓ |  |  |  |  |  |
|  |  |  | When I get bored I am unhappy |  | ✓ |  |  |  |  |  |  |  |  |  |  |  |  |  |
| Peer Network and Dyadic Loneliness Scale (PNDLS)^54^ | Hoza et al. (2000) | Participants are first asked to select which of the two types of children they are most like and then to specify whether the chosen description is sort of true or really true for them. | Some kids almost always feel left out when they’re with others their age BUT other kids almost never feel left out when they’re with others their age |  |  |  |  | ✓ |  | ✓ | ✓ |  |  |  |  |  |  |  |
|  |  |  | Some kids don’t have a friend they can talk to about important things BUT other kids do have a friends that they can talk to about important things |  |  |  |  | ✓ | ✓ |  |  |  |  |  |  |  |  |  |
|  |  |  | Some kids don’t have anyone special their age to share things with BUT other kids do have anyone special their age to share things with |  |  |  |  | ✓ | ✓ |  |  |  |  |  |  |  |  |  |
|  |  |  | Some kids feel like most kids like them BUT other kids feel like hardly any kids like them |  |  | ✓ |  | ✓ |  | ✓ | ✓ |  |  |  |  |  |  |  |
|  |  |  | Some kids feel like they really fit in with other kids BUT other kids don’t feel like they fit in very well with other kids |  |  | ✓ | ✓ | ✓ |  | ✓ |  | ✓ |  |  |  |  |  |  |
|  |  |  | Some kids feel lonely a lot because they wish other kids included them more in things BUT other kids don’t feel lonely very much because they think other kids usually do include them in things |  |  |  |  |  |  |  |  |  |  |  |  |  |  |  |
|  |  |  | Some kids hardly ever feel accepted by others their age BUT other kids feel accepted by others their age most of the time |  |  | ✓ |  | ✓ |  | ✓ | ✓ |  |  |  |  |  |  |  |
|  |  |  | Some kids hardly ever feel lonely because they have a best friend BUT other kids wish they had a best friend so they wouldn’t feel so lonely |  |  |  |  | ✓ | ✓ | ✓ |  |  |  |  |  |  |  |  |
|  |  |  | Some kids have a friend that is always there for them when they need it BUT other kids don’t have a friend that is always there for them when they need it |  |  |  |  | ✓ | ✓ |  |  |  |  |  |  |  |  |  |
|  |  |  | Some kids have a friend that they know will always care about them BUT other kids just with they had a friend that would always care about them |  |  |  |  | ✓ | ✓ |  |  |  |  |  |  |  |  |  |
|  |  |  | Some kids have someone their age who is really close friends BUT other kids don’t have anybody their age who is a really close friend |  |  |  |  | ✓ | ✓ |  |  |  |  |  |  |  |  |  |
|  |  |  | Some kids really feel like they’re part of a group BUT other kids feel like they’re not really part of a group |  |  |  |  | ✓ | ✓ | ✓ |  |  |  |  |  |  |  |  |
|  |  |  | Some kids usually have other kids to do things with BUT other kids hardly ever have kids to do things with |  |  |  |  | ✓ |  |  |  |  |  |  |  |  |  |  |
|  |  |  | Some kids wish they had a friend that really cared about how they feel inside BUT other kids feel like they already do have a friend that really cared about how they feel inside |  |  | ✓ |  | ✓ | ✓ |  |  |  |  |  |  |  |  |  |
| Rotenberg 3-item Loneliness and Social Dissatisfaction Measure^55^ | Rotenberg et al. (2004) | In the last two weeks, to what extent have you felt the following.  Options: never (1), sometimes (2), always (3). | I feel alone |  |  | ✓ |  |  |  | ✓ |  |  | ✓ |  |  |  |  |  |
|  |  |  | I feel left out |  |  | ✓ |  |  |  | ✓ | ✓ |  |  |  |  |  |  |  |
|  |  |  | I have no one to talk to |  |  |  |  | ✓ | ✓ | ✓ | ✓ |  |  |  |  |  |  |  |
| Relational Provisions Loneliness Questionnaire (RPLQ)^56^ | Hayden-Thomson (1989) | Participants rate how true each statement is for them on a 5-point Likert scale ranging from 1 (not at all true) to 5 (always true). | I feel in tune with other children |  |  | ✓ |  | ✓ |  |  |  |  |  |  |  |  |  |  |
|  |  |  | I feel in tune with the people in my family |  |  | ✓ |  | ✓ |  | ✓ |  |  |  |  |  |  |  |  |
|  |  |  | I feel like other children want to be with me |  |  | ✓ |  | ✓ |  | ✓ |  |  |  |  |  |  |  |  |
|  |  |  | I feel like people in my family want to be with me |  |  | ✓ |  | ✓ |  |  |  |  |  |  |  |  |  |  |
|  |  |  | I feel like that I usually fit in with other children around me |  |  | ✓ |  | ✓ |  | ✓ |  |  |  |  |  |  |  |  |
|  |  |  | I feel part of a group of friends that does things together |  |  |  |  | ✓ | ✓ | ✓ |  |  |  |  |  |  |  |  |
|  |  |  | I feel that I usually fit with my family |  |  | ✓ | ✓ | ✓ |  | ✓ |  |  |  |  |  |  |  |  |
|  |  |  | I have a friend I can tell everything to |  |  |  |  | ✓ | ✓ |  |  |  |  |  |  |  |  |  |
|  |  |  | I have a friend who is really interested in hearing about my private thoughts and feelings |  |  |  |  | ✓ | ✓ |  |  |  |  |  |  |  |  |  |
|  |  |  | I have a lot in common with other children |  |  |  | ✓ | ✓ |  | ✓ |  |  |  |  |  |  |  |  |
|  |  |  | I have at least one person in my family I can talk to when something is bothering me |  |  |  |  | ✓ | ✓ |  |  |  |  |  |  |  |  |  |
|  |  |  | I have at least one really good friend I can talk to when something is bothering me |  |  |  |  | ✓ | ✓ |  |  |  |  |  |  |  |  |  |
|  |  |  | I have someone in my family who is really interested in hearing about my private thoughts and feelings |  |  |  |  | ✓ | ✓ |  |  |  |  |  |  |  |  |  |
|  |  |  | I have someone in my family I can tell everything to |  |  |  |  | ✓ | ✓ |  |  |  |  |  |  |  |  |  |
|  |  |  | In my family, I feel part of a group of people that does things together |  |  |  |  | ✓ |  | ✓ |  |  |  |  |  |  |  |  |
|  |  |  | There is a friend I feel close to |  |  |  |  | ✓ | ✓ |  |  |  |  |  |  |  |  |  |
|  |  |  | There is somebody my age who really understands me |  |  |  |  | ✓ | ✓ |  |  |  |  |  |  |  |  |  |
|  |  |  | There is someone in my family I can turn to |  |  |  |  | ✓ | ✓ |  |  |  |  |  |  |  |  |  |
|  |  |  | There is someone in my family I could go to if I were feeling down |  |  |  |  | ✓ | ✓ |  |  |  |  |  |  |  |  |  |
|  |  |  | There is someone in my family I feel close to |  |  |  |  | ✓ | ✓ |  |  |  |  |  |  |  |  |  |
|  |  |  | There is someone in my family who really understands me |  |  |  |  | ✓ | ✓ |  |  |  |  |  |  |  |  |  |
|  |  |  | There is someone my age I can go to if I were feeling down |  |  |  |  | ✓ | ✓ |  |  |  |  |  |  |  |  |  |
|  |  |  | There is someone my age I can turn to |  |  |  |  | ✓ | ✓ |  |  |  |  |  |  |  |  |  |
|  |  |  | When I am with my family, I feel like I belong |  |  |  |  | ✓ |  |  |  |  |  |  |  |  |  |  |
|  |  |  | When I am with other children, I feel like I belong |  |  |  |  | ✓ |  | ✓ |  |  |  |  |  |  |  |  |
|  |  |  | When I want to do something for fun, I can usually find friends to join me |  |  |  |  | ✓ |  |  |  |  |  |  |  |  |  |  |
|  |  |  | When I want to do something for fun, I can usually find people in my family to join me |  |  |  |  | ✓ |  |  |  |  |  |  |  |  |  |  |
| Rasch-Type Loneliness Scale (RTLQ)/ De Jong Gierveld Loneliness Scale (DJGLS)^57^ | De Jong Gierveld & Kamphuis (1985) | Please indicate for each of the statements, the extent to which they apply to your situation, the way you feel now.  Options: yes!, yes, more or less, no, and no! | I can call on my friends whenever I need them |  |  |  |  | ✓ | ✓ |  |  |  |  |  |  |  |  |  |
|  |  |  | I find my circle of friends and acquaintances too limited |  |  | ✓ |  | ✓ |  |  |  |  |  |  |  |  |  |  |
|  |  |  | I miss having a really close friend |  |  |  |  | ✓ | ✓ |  |  |  |  |  |  |  |  |  |
|  |  |  | I miss having people around |  |  |  |  |  |  | ✓ |  |  |  |  |  |  |  |  |
|  |  |  | I miss the pleasure of the company of others |  | ✓ |  |  |  |  | ✓ |  |  |  |  |  |  |  |  |
|  |  |  | I often feel rejected |  |  | ✓ |  |  |  | ✓ | ✓ |  |  |  |  |  |  |  |
|  |  |  | There are enough people I feel close to |  |  |  |  |  | ✓ | ✓ |  |  |  |  |  |  |  |  |
|  |  |  | There are many people I can trust completely |  |  |  |  |  | ✓ |  |  |  |  |  |  |  |  |  |
|  |  |  | There are plenty of people that I can lean on when I have problems |  |  |  |  | ✓ | ✓ |  |  |  |  |  |  |  |  |  |
|  |  |  | There is always someone I can talk to about my day-to-day problems |  |  |  |  |  | ✓ |  |  |  |  |  |  |  |  |  |
| Social and Emotional Loneliness Scale for Adults (SELSA)^58^ | DiTommaso & Spinner (1993) | Participants are asked to indicate the degree of agreement or disagreement with each statement.  Options: 1 (strongly disagree), 2 (disagree), 3 (somewhat disagree), 4 (no label), 5 (somewhat agree), 6 (agree), and 7 (strongly agree). | I can depend upon my friends for help |  |  |  |  | ✓ | ✓ |  |  |  |  |  |  |  |  |  |
|  |  |  | I do not feel satisfied with the friends that I have |  | ✓ |  |  | ✓ | ✓ |  |  |  |  |  |  |  |  |  |
|  |  |  | I don't have a friend(s) who shares my views, but I wish I did |  |  | ✓ | ✓ | ✓ | ✓ |  |  |  |  |  |  |  |  |  |
|  |  |  | I don't have a friend(s) who understands me, but I wish I did |  |  |  |  | ✓ | ✓ |  |  |  |  |  |  |  |  |  |
|  |  |  | I feel 'in tune' with others |  |  | ✓ |  |  | ✓ | ✓ |  |  |  |  |  |  |  |  |
|  |  |  | I feel alone when I'm with my family |  |  | ✓ |  | ✓ | ✓ | ✓ |  |  | ✓ |  |  |  |  |  |
|  |  |  | I feel close to my family |  |  | ✓ |  | ✓ | ✓ |  |  |  |  |  |  |  |  |  |
|  |  |  | I feel part of a group of friends |  |  |  |  | ✓ |  | ✓ |  |  |  |  |  |  |  |  |
|  |  |  | I feel part of my family |  |  | ✓ |  | ✓ | ✓ | ✓ |  |  |  |  |  |  |  |  |
|  |  |  | I find myself wishing for someone with whom to share my life |  |  |  |  | ✓ | ✓ |  |  |  |  |  |  |  |  |  |
|  |  |  | I have a friend(s) with whom I can share my views |  |  |  | ✓ | ✓ | ✓ |  |  |  |  |  |  |  |  |  |
|  |  |  | I have a lot in common with others |  |  |  | ✓ |  | ✓ |  |  |  |  |  |  |  |  |  |
|  |  |  | I have a romantic or marital partner who gives me the support and encouragement I need |  |  |  |  | ✓ | ✓ |  |  |  |  |  |  |  |  |  |
|  |  |  | I have a romantic partner with whom I share my most intimate thoughts and feelings |  |  |  |  | ✓ | ✓ |  |  |  |  |  |  |  |  |  |
|  |  |  | I have an unmet need for a close romantic relationship |  |  |  |  | ✓ | ✓ |  |  |  |  |  |  |  |  |  |
|  |  |  | I have friends that I can turn to for information |  |  |  |  | ✓ |  |  |  |  |  |  |  |  |  |  |
|  |  |  | I have friends whom I can talk to about the pressures in my life |  |  |  |  | ✓ | ✓ |  |  |  |  |  |  |  |  |  |
|  |  |  | I have someone who fulfils my emotional needs |  |  |  |  | ✓ | ✓ |  |  |  |  |  |  |  |  |  |
|  |  |  | I have someone who fulfils my need for intimacy |  |  |  |  | ✓ | ✓ |  |  |  |  |  |  |  |  |  |
|  |  |  | I like the people I hang out with |  |  |  |  | ✓ |  |  |  |  |  |  |  |  |  |  |
|  |  |  | I really belong in my family |  |  |  |  | ✓ | ✓ | ✓ |  |  |  |  |  |  |  |  |
|  |  |  | I wish I had a more satisfying romantic relationship |  |  |  |  | ✓ | ✓ |  |  |  |  |  |  |  |  |  |
|  |  |  | I wish my family was more concerned about my welfare |  |  | ✓ |  | ✓ | ✓ |  |  |  |  |  |  |  |  |  |
|  |  |  | I'm not part of a group of friends and I wish I were |  |  |  |  | ✓ |  | ✓ |  |  |  |  |  |  |  |  |
|  |  |  | My family really cares about me |  |  | ✓ |  | ✓ | ✓ |  |  |  |  |  |  |  |  |  |
|  |  |  | My friends understand my motives and reasoning |  |  |  |  | ✓ | ✓ | ✓ |  |  |  |  |  |  |  |  |
|  |  |  | No one in my family really cares about me |  |  |  |  | ✓ | ✓ | ✓ |  |  |  |  |  |  |  |  |
|  |  |  | There is no one in my family I can depend upon for support and encouragement, but I wish there were |  |  | ✓ |  | ✓ | ✓ |  |  |  |  |  |  |  |  |  |
|  |  |  | There is no one in my family I feel close to, but I wish there were |  |  | ✓ |  | ✓ | ✓ |  |  |  |  |  |  |  |  |  |
|  |  |  | What's important to me doesn’t seem to be important to the people I know |  |  | ✓ | ✓ |  | ✓ |  |  |  |  |  |  |  |  |  |
| Single-item^59^ | Bayat et al. (2021) | Options: “yes, often”, “yes, sometimes”, “seldom”, and “no”. | Do you ever feel lonely? |  |  |  |  |  |  | ✓ |  |  |  |  |  |  |  |  |
| Single-item^60^ | Reinwarth et al. (2023) | Options: 0 = “no, does not apply”, 1 = “yes, it applies, but I do not suffer from it”, 2 = “yes, it applies, and I suffer slightly”, 3 = “yes, it applies, and I suffer moderately”, 4 = “yes, it applies, and I suffer strongly” | I am frequently alone/have few contacts |  |  |  |  | ✓ |  |  |  |  | ✓ |  |  |  |  |  |
| University of California Los Angeles (UCLA) Loneliness Scale^61^ | Russell et al. (1980) | Indicate how often you feel the way described in each of the following statements.  Options: never (1), rarely (2), sometimes (3), often (4). | I am no longer close to anyone |  |  |  |  |  | ✓ |  |  |  |  |  |  |  |  |  |
|  |  |  | I can find companionship when I want it |  |  |  |  | ✓ |  |  |  |  |  |  |  |  |  |  |
|  |  |  | I do not feel alone |  |  | ✓ |  |  |  | ✓ |  |  | ✓ |  |  |  |  |  |
|  |  |  | I feel in tune with the people around me |  |  | ✓ | ✓ |  | ✓ | ✓ |  |  |  |  |  |  |  |  |
|  |  |  | I feel isolated from others |  |  | ✓ |  |  |  |  |  |  | ✓ |  |  |  |  |  |
|  |  |  | I feel left out |  |  | ✓ |  |  |  | ✓ | ✓ |  |  |  |  |  |  |  |
|  |  |  | I feel part of a group of friends |  |  |  |  | ✓ |  |  |  |  |  |  |  |  |  |  |
|  |  |  | I have a lot in common with the people around me |  |  |  | ✓ |  |  | ✓ |  |  |  |  |  |  |  |  |
|  |  |  | I lack companionship |  |  |  |  | ✓ | ✓ | ✓ |  |  |  |  |  |  |  |  |
|  |  |  | My interests and ideas are not shared by those around me |  |  |  | ✓ | ✓ | ✓ | ✓ |  |  |  |  |  |  |  |  |
|  |  |  | My social relationships are superficial |  |  |  |  |  | ✓ |  |  |  |  |  |  |  |  |  |
|  |  |  | No one really knows me well |  |  |  |  |  | ✓ |  |  |  |  |  |  |  |  |  |
|  |  |  | People around me but not with me |  |  |  |  | ✓ | ✓ | ✓ |  |  | ✓ |  |  |  |  |  |
|  |  |  | There are people I can talk to |  |  |  |  | ✓ | ✓ |  |  |  |  |  |  |  |  |  |
|  |  |  | There are people I can turn to |  |  |  |  |  | ✓ |  |  |  |  |  |  |  |  |  |
|  |  |  | There are people I feel close to |  |  |  |  |  | ✓ | ✓ |  |  |  |  |  |  |  |  |
|  |  |  | There are people who really understand me |  |  |  |  |  | ✓ | ✓ |  |  |  |  |  |  |  |  |
|  |  |  | There is no one I can turn to |  |  |  |  |  | ✓ |  |  |  |  |  |  |  |  |  |
